# Conditions of the Presence of Bimodal Amplitude Distribution of Two-Process Surfaces

**DOI:** 10.3390/ma13184037

**Published:** 2020-09-11

**Authors:** Pawel Pawlus, Rafal Reizer, Michal Wieczorowski

**Affiliations:** 1 Faculty of Mechanical Engineering and Aeronautics, Rzeszow University of Technology, Powstancow Warszawy 8 Street, 35-959 Rzeszow, Poland; ppawlus@prz.edu.pl; 2 College of Natural Sciences, University of Rzeszow, Pigonia Street 1, 35-310 Rzeszow, Poland; 3 Faculty of Mechanical Engineering and Management, Poznan University of Technology, Piotrowo Street 3, 61-138 Poznan, Poland; michal.wieczorowski@put.poznan.pl

**Keywords:** two-process surface, bimodal distribution, material ratio, parameters

## Abstract

Two-process surfaces are functionally important. They contain plateau and valley parts. They are created by superimpositions of two one-process textures of Gaussian probability height distributions. It is expected that the resulting two-process surface would have bimodal height probability distribution. However, typically two-process textures have unimodal ordinate distribution. The present authors developed limiting conditions of presence of bimodal ordinate distribution. These conditions depend on the material ratio at the plateau-to-valley transition (the Smq parameter), and on the ratio of heights of the plateau and valley surface parts (Spq/Svq). Generated stratified textures and measured two-process surfaces of cylinder liners were taken into consideration.

## 1. Introduction

Surface topography is the fingerprint of a manufacturing process. It affects functional properties of machine elements, such as contact, sealing, friction, and wear [1]. Typically, one-process random surfaces are taken into consideration. Some natural and engineering surfaces have self-affine properties. The fractal analysis should be applied carefully. Not all random surfaces exhibit self-affine properties. During a low wear (within the limits of the machined surface topography), the one-process surface changes and a two-process surface is created. Previously, a surface subjected to low wear was simulated by truncation of the peaks positioned above a given threshold parallel to the mean plane [2,3,4,5]. However, this performance seldom occurs. In practice, the obtained transitional surface topography consists of the machined surface and of created during the wear a fine surface with a Gaussian height probability ordinate distribution. This fine surface became smoother as wear progressed [6,7]. Tribological properties of two-process surfaces were found to be better than those of one-process textures [8,9,10]. Therefore, two-process surfaces were machined. The plateau-honed cylinder surface is the practical example of machined two-process textures [4,7,11,12]. The stratified property of the two-process surface substantially affects the contact behavior [13,14,15,16,17,18]. The contact stiffness and the normal deformation are governed by the fine part of two-process surface.

The analysis of multi-process textures is more difficult than the study of one-process surfaces. Parameters frequently used to describe one-process surface cannot be applied for two-process topography, such as cylinder liner surface after plateau honing [19]. Many parameters are related to the material ratio curve, which presents the cumulative distribution of surface ordinates. The horizontal axis characterizes the bearing (material) ratio and the vertical axis characterizes the depth. According to ISO 13565-2 standard a surface consists of three parts: core (central), peaks, and valleys. Therefore, three parameters are applied to characterize the heights: core roughness depth which describes the central part of the material ratio curve, reduced peak height and reduced valley depth. The division between zones is performed by sliding 40% of material ratio wide window through the material ratio curve and finding the minimum secant slope [20,21,22,23].

**Figure 1:**
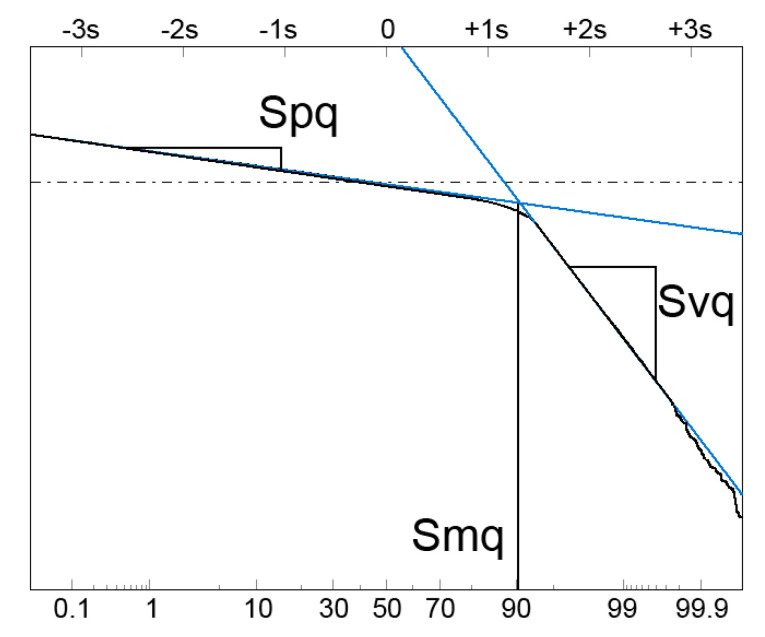
Graphical interpretation of the probability parameters: Spq, Svq and Smq.

In the other approach, based on material probability curve (ISO 13565-3 standard), two parts of two-process surface appear: plateau (peak) and valley [24,25,26,27]. Material probability curve is a representation of the material ratio curve; the material ratio is expressed as Gaussian probability in standard deviation values, plotted linearly on the horizontal axis (−3s = 0.13%, −2s = 2.28%, −s = 15.8%, 0 = 50%, s = 84.13%, 2s = 97.72%, 3s = 99.87%). The probability plot of two-process surface presents two straight non-parallel lines. The Spq parameter is the rms. height of the plateau part and the Svq parameter is the rms. height of the valley part. The Spq and Svq parameters are the slopes of the linear regressions through the plateau and valley parts, respectively. This approach contains also the Smq parameter, which is the material ratio at the transition point between plateau and valley parts—Figure 1. Since these parameters are statistical, similar values can be obtained for 2D profile and 3D (areal) texture. Because this method is based on theoretical presumptions, it can be applied in multi-process surface modeling. This material ratio curve is related to tribological properties of functional elements such as a load-carrying capacity and a wear resistance [28].

During the creation of a two-process random surface, peak (plateau) Gaussian surface is superimposed on the valley Gaussian surface. It is expected that two-process surface would have bimodal ordinate distribution. A bimodal distribution is a probability distribution having two different modes; distinct local maxima exist in the height probability density function. However, the probability distribution of two-process texture can be also unimodal. The conditions of bimodal amplitude distribution of two-process surface should be developed.

## 2. Theoretical Considerations

**Figure 2:**
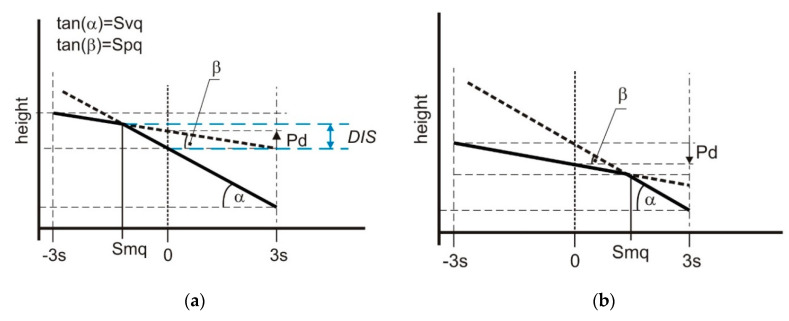
Probability plots of material ratio curves for different two-process surfaces; the Smq parameter is smaller than 50% (**a**) and the Smq parameter is higher than 50% (**b**) with plateau depth Pd and the vertical distance between two modes DIS.

Figure 2 presents two different cases of probability plots of material ratio curves of two-process surfaces. In Figure 2a the Smq parameter is smaller than 50% (negative values of the standard deviation s). However, in Figure 2b the Smq parameter is higher than 50% (positive values of s). The plateau depth Pd, which is the distance between the mean planes of two Gaussian surfaces: plateau and valley, is also shown in Figure 2.

The parameters Spq, Svq, Smq are related to the plateau depth Pd (Figure 2) by the following relation:

Pd = Smq·(Spq − Svq),(1)

Description of the probability plot of the material ratio curve is related to computer generation of two-process surfaces, which relies on superimposition of two Gaussian textures. The first of them (plateau) is characterized by the standard deviation of height Sq equal to the Spq parameter of a two process surface, while the Sq parameter of the second (valley) texture is equal to the Svq parameter of the two-process surface. The vertical distance between mean planes of these Gaussian structures is the plateau depth Pd. It should be noted that the rms. height Sq does not completely characterize each Gaussian surface, the correlation length CL (the distance, at which the autocorrelation function decays to 0.1 value) must be specified. For an isotropic surface, the correlation lengths in perpendicular directions are the same. From two Gaussian surfaces, smaller ordinates are chosen to generate two-process texture. This method was described in detail in [29]. The authors of papers [30,31,32,33] used similar procedures. Of course, generation of two-process surface structures across multiple scales is a further topic that requires attention.

**Figure 3:**
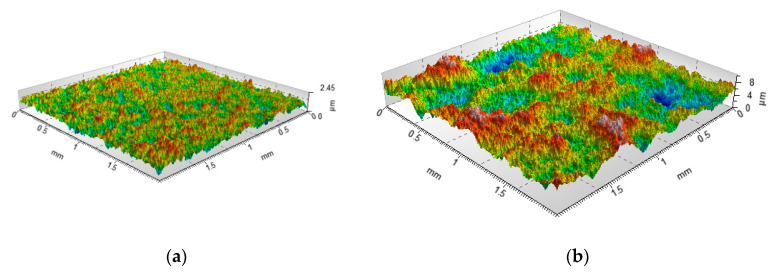

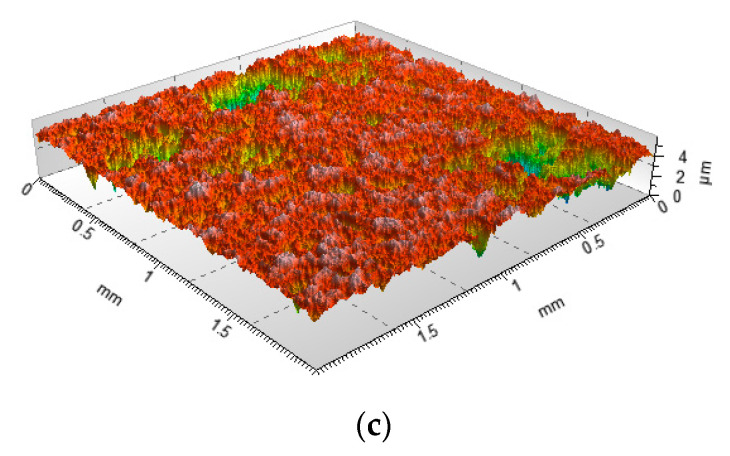
Example of generation of two-process surface topography: the plateau surface: Sq = 0.3 µm, CL = 20 µm (**a**), the valley surface: Sq = 1.7 µm, CL = 150 µm (**b**), two-process surface: Spq = 0.3 µm, Svq = 1.7 µm, Smq = 72.7% (**c**), Sq is the rms. height, CL is the correlation length.

Figure 3 presents an example of a computer generated two-process surface.

During creation of two-process surface, typically two Gaussian surfaces are superimposed; therefore, two-process texture can be called bi-Gaussian surface. However, the plateau surface is random, but the valley surface can be random or deterministic. Therefore, the two-process surface is a more general expression. Sometimes, multi-process textures are created, for example during wear of plateau-honed cylinder surface. However, in this paper two-process textures are analyzed.

Bimodal probability distribution of a two-process surface is only possible when the Smq parameter is smaller than 0 (50%) (Figure 2a). In the other case (Figure 2b), the Smq parameter is higher than 0 (50%), the modal value of the valley part is located above the modal value of the plateau part, so unimodal distribution is obtained; the peak (maximum) corresponds to the material ratio of 50%. Unimodal height probability also takes place when the Smq parameter is equal to 50%—when the means of the two normal distributions are equal, and the combined distribution is unimodal.

For bimodal probability height distribution (Figure 2a) the lower mode corresponds to the material ratio of 50% (s = 0), however, the upper mode corresponds to the Smq parameter. The vertical distance between two modes should be:

DIS = Pd − Spq·Smq = −Smq·Svq,(2)

However, sometimes when DIS is higher than 0, only unimodal distribution can be obtained, particularly when the modes of two Gaussian surfaces are close to each other. The conditions of presence of bimodal distribution will be developed in this work.

The vertical position of the maximum value of the probability height distribution corresponds to the smallest slope of the material ratio curve–inflection point, which is probably tribologically important.

## 3. The Analysis of Generated Surfaces

Two-process random isotropic topographies were generated. The superimposition method was used. Each Gaussian surface was modeled using procedure developed by Wu [34]. Each surface contained 256 × 256 points. The sampling interval was 1 µm, the correlation lengths in perpendicular directions were 10 µm. The Svq/Spq ratios were in the range: 4–30.

The aforementioned assumptions in Section 2 were confirmed. The upper peak of the bimodal probability distribution corresponded to the material ratio at the transition point Smq. The lower peak corresponded to the material ratio of 50%. Therefore, the vertical distance between two modes DIS were equal to −Smq·Svq. The mean error was 1.8%. This distance was typically higher that the plateau depth Pd. The average relative difference between the vertical distance between two modes DIS and the plateau depth Pd was 19%. This difference was smaller for higher value of the Smq material ratio and also for higher values of the Svq/Spq ratio.

**Figure 4:**
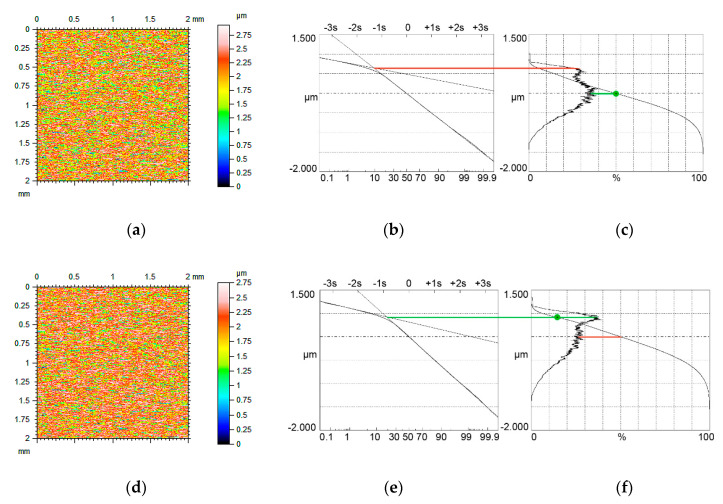
Contour plots (**a**,**d**) surface probability plot (**b**,**e**), material ratio curve and probability distribution (**c**,**f**) of two-process isotropic modeled surfaces of the Smq parameter of 10% (**a**–**c**) and 20% (**d**–**f**), the other parameters of both surfaces are Spq = 0.12 µm, Svq = 0.5 µm, both surfaces have bimodal ordinate distribution.

**Figure 5:**
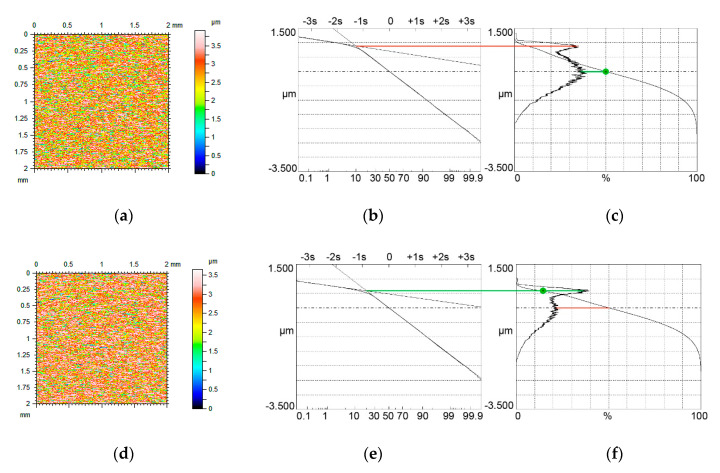
Contour plots (**a**,**d**) surface probability plot (**b**,**e**), material ratio curve and probability distribution (**c**,**f**) of two-process isotropic modeled surfaces of the Smq parameter of 10% (**a**–**c**) and 20% (**d**–**f**), the other parameters of both surfaces are Spq = 0.12 µm, Svq = 0.7 µm, both surfaces have bimodal ordinate distributions.

**Figure 6:**
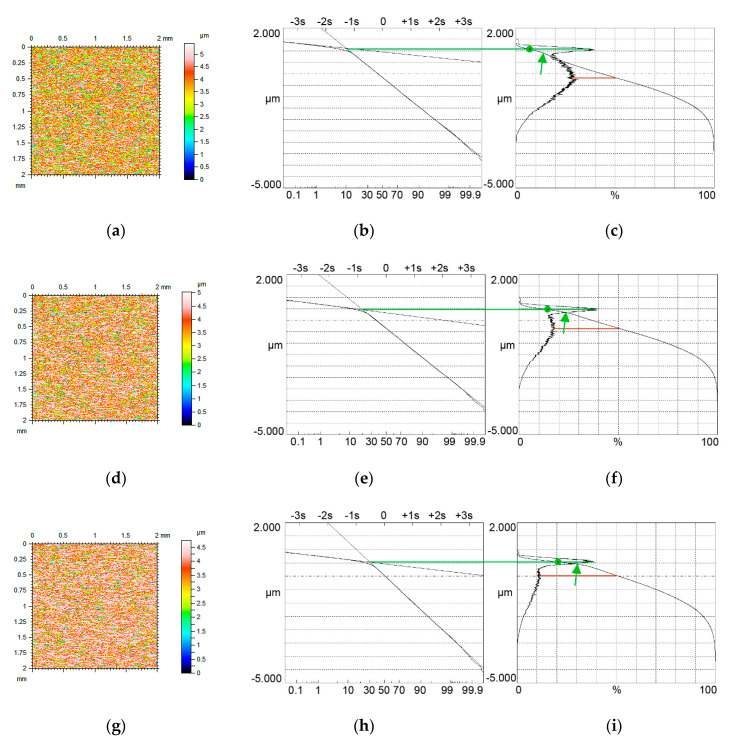
Contour plots (**a**,**d**,**g**) surface probability plot (**b**,**e**,**h**), material ratio curve and probability distribution (**c**,**f**,**i**) of two-process isotropic modeled surfaces of the Smq parameter of 10% (**a**–**c**), 20% (**d**–**f**) and 30% (**g**–**i**), the other parameters of both surfaces are Spq = 0.12 µm, Svq = 1.0 µm, all surfaces have bimodal ordinate distributions.

Figure 4, Figure 5 and Figure 6 present contour plots of computer-generated surfaces, their probability plots of material ratio curves, and material ratio curves with probability height distributions. The larger mode is called the major mode and the other mode is called the minor mode. The green line shows the position of the major mode while the red line shows the location of the minor mode. In addition, the smallest slope of the material ratio curve is marked by a green circle.

The major mode corresponds to the smallest slope of the material ratio curve. Typically, the upper peak (local maximum) is the major mode. However, for small values of the Svq/Spq ratio, and for low material ratio at the transition point Smq, the lower peak can be the major mode (Figure 4c). The ratio of the amplitudes of higher and lower peaks is called the bimodal ratio [35]. When the Smq parameter increased, the bimodal ratio also increased and the vertical distance between two modes DIS decreased.

When the Smq material ratio was 10% and the Svq/Spq ratio was 4.17, the smallest slope of the material ratio curve was obtained for the material ratio of 50% (green circle in Figure 4c). Due to an increase in the Smq parameter to 20%, the smallest slope of the material ratio curve corresponded to the Smq parameter. Owing to an increase in the Smq material ratio, the skewness Ssk decreased (from −0.35 to −0.57). When the Smq parameter further increased to 25%, only one peak was visible in the ordinate distribution—unimodal distribution took place.

A similar situation occurred when the Svq parameter increased to about 0.7 µm (Figure 5). The amplitudes of both modes are similar for the Smq parameter of 10% (Figure 5c); therefore, the bimodal ratio increased compared to Figure 4c. This ratio is also higher in Figure 5f, compared to Figure 4f, when the Smq parameter increased to 20%. When the Smq parameter increased to 25%, the amplitude probability distribution was still bimodal, but when Smq was 30% unimodal distribution occurred. Similar to lower Svq/Spq ratio, the smallest slope of the material ratio curve corresponded to the material ratio of 50% for the Smq parameter of 10% and to the material ratio of 20% when the Smq parameter increased.

When the Svq/Spq ratio further increased to a value of 8.3 (Figure 6) the upper peak was the major mode even for the Smq parameter of 10%. The bimodal ratio increased for the same Smq parameter compared to textures shown in Figure 4 and Figure 5. Bimodal distribution existed even for the Smq parameter near 30%. One can find in Figure 6 points in material ratio curves, which correspond to changes from the plateau parts to the valley surface portions. In these points, the first derivatives are discontinuous. These points, indicated by green arrows, are located under the major mode. Because the tribological properties of surfaces depend on the material ratio curve, the presence of those points can have functional significance. The described points were also visible for higher Svq/Spq ratios.

The question arises: what is the limiting condition for presence of bimodal height probability distribution? Conditions of unimodality or bimodality depend typically on the height standard deviations of two Gaussian height distributions [35,36,37,38,39,40]. Near the transition point of the Smq material ratio, there is a mixture of two Gaussian distributions. The analysis of many generated two-process surfaces revealed that this mixture also depends on the height standard deviations of two distributions. When the Svq parameter is much higher than the Spq parameter, the following condition of bimodal height probability distribution presence was established:

DIS > Spq + 0.5·Svq,(3)

**Figure 7:**
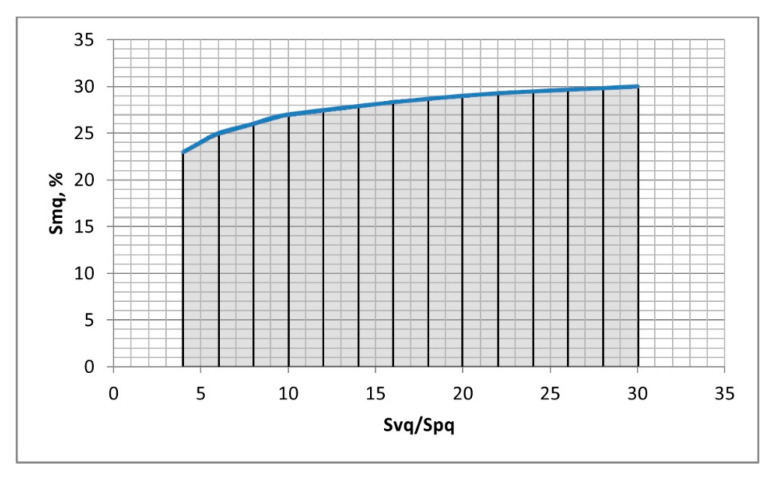
Conditions of bimodal height distributions of two-process surfaces; bimodal distribution appears for the Smq parameter within the shaded area.

It was found from the analysis of simulated surfaces that this condition was valid for the Svq/Spq ratio not smaller than 4. The highest analysed Svq/Spq ratio in this work was 30. On the basis of the analysis of many modeled two-process textures it was found that the limiting condition of presence of bimodal ordinate distribution depended on the Svq/Spq ratio. The probability height distribution of a two-process surface is bimodal when the Smq material ratio is lower than the values shown in Figure 7. The shaded area corresponds to bimodal height distribution. One can see that the limiting value of the Smq ratio is higher when the Svq/Spq ratio is higher. This dependence is stronger for smaller Svq/Spq ratio. It was also found that unimodal ordinate distribution occurred for the Smq parameter was higher than the values presented in Figure 7.

**Figure 8:**
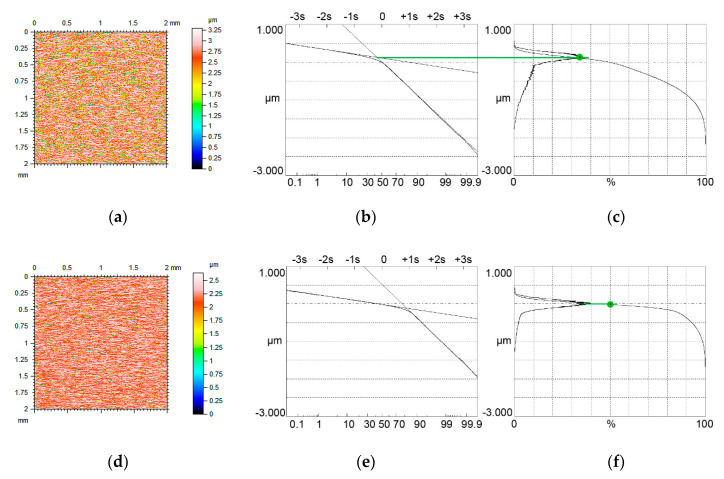
Contour plots (**a**,**d**) surface probability plot (**b**,**e**), material ratio curve and probability distribution (**c**,**f**) of two-process isotropic modeled surfaces of the Smq parameter of 40% (**a**–**c**) and 80% (**d**–**f**), the other parameters of both surfaces are Spq = 0.12 µm, Svq = 0.7 µm, both surfaces have unimodal ordinate distributions.

When the Smq parameter is higher than that presented in Figure 7, but smaller than 50% then only one mode exists for the Smq material ratio (Figure 8a–c).

However, when the Smq parameter is similar or higher than 50% the mode corresponds to the material ratio of 50% (Figure 8d–f). The assumptions presented in Section 2 were confirmed for all modeled textures.

The presented analysis can be extended for multi-process surfaces, when the number of processes is higher than 2, especially for three-process structures. The material ratio at the transition point between the second and the third part would be very small for three-modal ordinate distribution. If the number of processes were higher than 3, the analysis would be more difficult.

## 4. The Analysis of Measured Surfaces

Cylinder surfaces after plateau honing are the typical examples of two-process textures. However, these cylinders are characterized by the Smq parameter higher than 50%, so their probability distributions are unimodal. Cylinder liners with bimodal surface topography were created during tests using an Optimol SRV5 (produced by: Optimol Instruments Prüftechnik GmbH, München, Germany) oscillating wear tester under lubricated conditions at high and low temperatures. This tester allows for precise control of the normal load, temperature and stroke. A chromium-coated piston rings were in contact with cylinder liners. These liners were initially one-process honed by diamond tools. One can find the operating conditions in References [41,42]. These surfaces were measured by a white light interferometer Talysurf CCI Lite (produced by: Taylor Hobson Ltd., Leicester, UK). The measuring area 3.29 × 3.29 mm^2^ contained 1024 × 1024 data points. Forms were removed by polynomials of the second degree. A digital filtration was not used.

**Figure 9:**
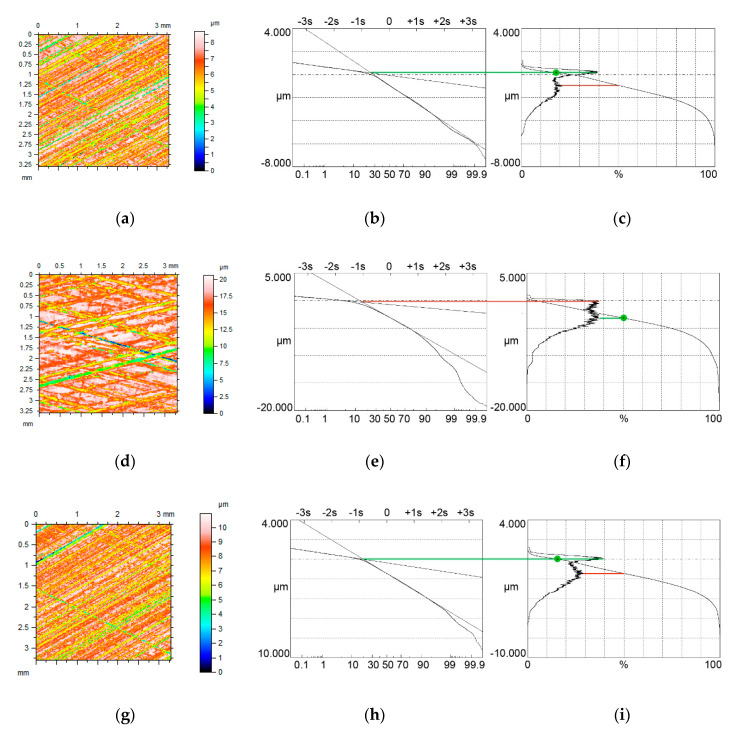
Contour plots (**a**,**d**,**g**) surface probability plot (**b**,**e**,**h**), material ratio curve and probability distribution (**c**,**f**,**i**) of two-process measured surfaces characterized by the following parameters: Spq = 0.31 µm, Svq = 1.61 µm, Smq = 24% (**a**–**c**), Spq = 0.49 µm, Svq = 2.89 µm, Smq = 15% (**d**–**f**) and Spq = 0.43 µm, Svq = 1.69 µm, Smq = 20% (**g**–**i**), three surfaces have bimodal ordinate distributions.

**Figure 10:**
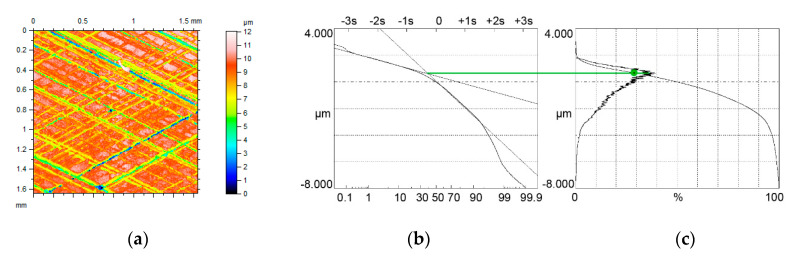

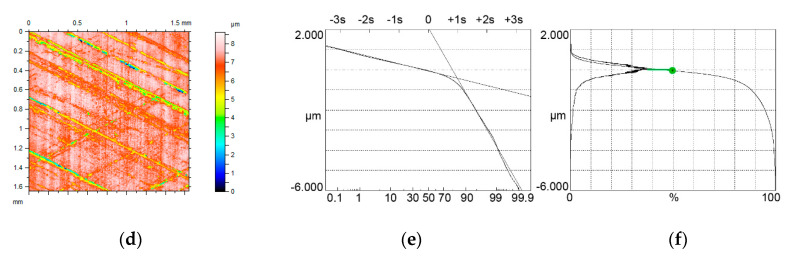
Contour plots (**a**,**d**) surface probability plot (**b**,**e**), material ratio curve and probability distribution (**c**,**f**) of two-process measured surfaces characterized by the following parameters: Spq = 0.61 µm, Svq = 2.04 µm, Smq = 38% (**a**–**c**) and Spq = 0.43 µm, Svq = 2.51 µm, Smq = 84%, both surfaces have unimodal ordinate distributions.

Figure 9 and Figure 10 present contour plots of computer-generated surfaces, their probability plots of material ratio curves and material ratio curves with probability height distributions.

The surface shown in Figure 9a–c was tested in high temperature (80 °C). The Svq/Spq ratio was higher than 5. The upper peak was the major mode, therefore this mode corresponded to the Smq material ratio of 24%. The second and the third surfaces were tested in negative temperature (−20 °C). The Svq/Spq ratio of the surface shown in Figure 9d–f was near 6. The Svq parameter of this surface was comparatively high (2.89 µm). However, due to a low value of the Smq parameter, the lower peak became the major mode and therefore this mode occurred for the material ratio of 50%. The Svq/Spq ratio of the third surface shown in Figure 9g–i was the smallest (near 4). However, due to the material ratio at the transition point equal to 20%, the upper peak was the major mode, the position of which corresponded to the Smq parameter.

When the Smq parameter was higher than 30%, cylinder liner textures had unimodal probability height distribution. Similar to modeled surfaces, when the Smq parameter was smaller than 50%, the mode appeared for the material ratio of Smq (Figure 10a–c). In the other cases, such as that shown in Figure 10d–f, this mode corresponded to the material ratio of 50%.

Generally, the assumptions presented in Section 2 were confirmed for measured surfaces. In addition, Equation (3) was found to properly discriminate between bimodal and unimodal amplitude probability distributions.

In this work we analyzed the vertical position of the major mode of amplitude distribution, which is also the position of the smallest slope of the material ratio curve of two-process surface. This point is of substantial practical significance, since the material ratio curve is strongly related to tribological properties of machine elements, such as a load-carrying capacity and a wear resistance. Perhaps the deformation at the smallest slope of the material ratio curve would be very low, which may be related to high wear resistance. Therefore, the results of this work are tribologically important.

For example, it was found [6,7] that during low wear the Spq parameter decreased and the Smq parameter increased. Therefore, one can predict that during wear of initial one-process random surface, initially the major mode and, perhaps more importantly, the smallest slope of the material ratio curve would be obtained for the material ratio of 50%. Then, during wear, the smallest slope material ratio would be decreased to the Smq parameter of new created two-process surface. Then during the test, the material ratio of the smallest slope would be increased to 50%, and it would be still 50% even for further Smq parameter increases. This prediction is important from a tribological point of view. Of course, during low wear, the smallest slope of the material ratio curve was reduced as the test progressed.

The findings obtained for areal 3D surface texture are also valid for 2D profile. However, due to the higher number of measuring points, the analysis of amplitude probability distribution of areal surface topography is easier compared to that of the 2D profile.

## 5. Conclusions

1. Limiting conditions of bimodal height distribution of two-process surface topography were developed. They depend on the ratio of the standard deviations of the valley and plateau parts Svq/Spq and on the material ratio at the transition between plateau and valley portions Smq. Based on these conditions, bimodal and unimodal height probability distributions were correctly discriminated for modeled and measured surfaces.
2. The bimodal ratio increased when the Svq/Spq ratio increased. Typically, the upper peak is the major mode. However, for low values of the Smq parameter and for low Svq/Spq ratio, the lower peak, which corresponds to the material ratio of 50%, can be the major mode.
3. When the Smq parameter is not lower than 50%, unimodal amplitude distribution exists. The mode and the smallest slope of the material ratio curve appear at the material ratio of 50%.
4. For unimodal height distribution and the value of the Spq parameter smaller than 50%, the mode corresponds to the Smq material ratio.
5. The results are functionally important because of the high tribological significance of the material ratio curve. In particular, the position of its smallest slope deserves attention.
